# Voluntary Out-of-Body Experience: An fMRI Study

**DOI:** 10.3389/fnhum.2014.00070

**Published:** 2014-02-10

**Authors:** Andra M. Smith, Claude Messier

**Affiliations:** ^1^School of Psychology, University of Ottawa, Ottawa, ON, Canada

**Keywords:** body representation, cerebellum, kinesthetic imagery, motor imagery, out-of-body experiences, somatosensory systems, temporal parietal junction

## Abstract

The present single-case study examined functional brain imaging patterns in a participant that reported being able, at will, to produce somatosensory sensations that are experienced as her body moving outside the boundaries of her physical body all the while remaining aware of her unmoving physical body. We found that the brain functional changes associated with the reported extra-corporeal experience (ECE) were different than those observed in motor imagery. Activations were mainly left-sided and involved the left supplementary motor area and supramarginal and posterior superior temporal gyri, the last two overlapping with the temporal parietal junction that has been associated with out-of-body experiences. The cerebellum also showed activation that is consistent with the participant’s report of the impression of movement during the ECE. There was also left middle and superior orbital frontal gyri activity, regions often associated with action monitoring. The results suggest that the ECE reported here represents an unusual type of kinesthetic imagery.

## Introduction

The experience of one’s body is a central process to allow us to interact with the outside world. Body experience is based on the integration of visual, vestibular, and somatosensory information (Giummarra et al., 2008; Berlucchi and Aglioti, 2010; de Vignemont, 2011; Blanke, 2012; Moseley et al., 2012). This information allows the tracking of the body in space and in relation with other objects and beings in our environment. Tracking of our body in turn, guides our movements (Goodale et al., 2008). The conscious experience of our body is generally congruent across sensory modalities so that, what we see of our body is also what we feel from somatosensory and vestibular sensations (Tsakiris, 2010). The sensations and percept associated with our body in movement can also be elicited in our imagination albeit most of the time in an attenuated form. Motor imagery corresponds to the cognitive version of motor actions without actual motor movements (Guillot et al., 2012; Moran et al., 2012). This motor “imagery” encompass visual components when we imagine movements as we would see them from our own perspective or from a third-person perspective (imagine someone else moving – or imagine ourselves moving but from a third-person perspective) and proprioceptive and vestibular components often referred to kinesthetic “imagery” (Guillot et al., 2009, p. 698). Motor imagery is intertwined within the brain’s preparatory processes preceding action and, up to a certain point, the brain’s processes subserving actual movement (Guillot and Collet, 2005). The strongest support for this view has come from functional imaging that demonstrated strong but incomplete overlap between imagery, action preparation, and action (Porro et al., 1996; Guillot et al., 2008, 2009; Szameitat et al., 2012a,b). These studies show that motor imagery is dependent both on brain regions associated with the performance of motor action but also on the somatosensory brain regions associated with body perception. Voluntary and involuntary motor imagery is also present in amputated individuals with an associated phantom limb often together with somatosensory perception (Melzack, 1989, p. 657; Ramachandran and Hirstein, 1998, p. 493). Some amputees can also train themselves to experience an anatomically impossible movement with their phantom limb suggesting the plasticity of sensorimotor systems (Moseley and Brugger, 2009, p. 1069).

The multi-component nature of body representation is also revealed in perceptual illusions such as the rubber hand illusion (Botvinick and Cohen, 1998). In the rubber hand illusion, the vision-based belief that the rubber hand is not part of the participant’s body is countered by the simultaneous touching of the rubber hand and the real hand and leads to a shift in the attribution of the localization of sensory stimulation from the real hand to the rubber hand (Hohwy and Paton, 2010). During the process of establishing the illusion, from completely separate to unity with the rubber hand, several intermediate illusory experiences can take place (Valenzuela Moguillansky et al., 2013, p. 1001). In one experiment using a moveable hand model, conditions could be manipulated so that participants reported a dissociation of the sense of ownership (impression that the fake hand is their own) or the sense of agency (impression that participants controlled the movements of the fake hand) (Kalckert and Ehrsson, 2012). Mismatch between the observed position of the hand model and the sensed position of the real hand reduced sense of ownership but did not disrupt the impression of agency. Conversely, passive movement reduced agency but left ownership intact (Kalckert and Ehrsson, 2012). These observations suggest that agency and ownership may depend on different but overlapping brain networks (Jackson et al., 2006, p. 703). Another experiment demonstrated that concurrent limb and full-body orientation illusions elicited by virtual reality visual displacement were undissociated and not dependent on action (Olive and Berthoz, 2012, p. 1050).

During these illusions, the participants do not doubt that the shifted body perception is illusory (Blanke and Metzinger, 2009). In contrast, shifted body perception of neurological origin (Blanke and Mohr, 2005) or pharmacologically induced (Morgan et al., 2011; Wilkins et al., 2011) can lead to ambiguous embodiment whereas people report that the illusory body or body part is more realistic or corresponds to a “double” of their body. In the descriptions below, the “double” refers to the illusory body (or parts thereof). There seems to be a general consensus in adopting the classification proposed by Brugger to describe these illusions (Brugger and Regard, 1997). Autoscopic hallucination is a visual hallucination of the upper part of a double of the body. Heautoscopy is a visual and somesthetic hallucination. The double, which appears as through a veil, can mirror the person’s movements. Heautoscopy hallucination is also accompanied by a vague feeling of detachment and depersonalization. The double is felt vaguely as another self. Feeling of a presence is a mostly somesthetic hallucination that a double is present usually close by or even touching but not seen. Feeling of a presence is also called sensed-presence experience when the presence is identified as another person (Cheyne and Girard, 2007, p. 1065). Out-of-body experience is a visual and somesthetic experience in which the double is seen from a different perspective, often motionless. Because the body in this experience is “seen” from a third-person perspective (i.e., from above), the body seen is illusory even if it is congruent with the body’s position during the illusion (e.g., lying down). The experience is accompanied by a profound feeling of being outside of the body and with feelings of meaningfulness of the experience.

Three studies of self-reported anomalous body experiences in unremarkable normal people (Braithwaite et al., 2011, p. 876; Braithwaite et al., 2011, p. 1063; Braithwaite et al., 2013, p. 1064). In the first one, it was noted that most instances of spontaneous anomalous body experiences occurred during a relaxed or borderline sleeping state and one-third reported (seeing) their body from a different perspective while the rest reported a visual or somatosensory shift in perspective. The participants who reported out-of-body experience also self-reported more perceptual anomalies (Braithwaite et al., 2011, p. 876). In two subsequent experiments, participants self-reporting anomalous body experiences (mostly of visual nature) were more likely to respond strongly to aversive visual patterns suggesting that the visual system of the participants are somehow different, at least functionally (Braithwaite et al., 2013, p. 1064; Braithwaite et al., 2013, p. 1063). The authors also derived the hypothesis that these anomalous body experiences depended on temporal lobe anomalies as measured by perceptual tasks and questionnaires (Braithwaite et al., 2011, p. 876).

There also have been imaging enquiries into the brain areas involved in body representation illusions in neurologically intact participants (Blanke, 2012). Brain imaging studies have suggested that activity in sensory integration areas such as the intraparietal sulcus and the ventral premotor cortex are associated with the establishment of the rubber hand illusion (Ehrsson et al., 2004, 2005, 2007; Tsakiris et al., 2007). One experiment has used repeated transcranial magnetic stimulation to gain information on the brain areas involved in the rubber hand illusion (Tsakiris et al., 2008). They found that, when the activity of the temporal parietal junction (TPJ) was perturbed by repeated transcranial magnetic stimulation, the processing of body representation mental imagery was impaired. However, in another transcranial magnetic stimulation study, mental rotation of letter stimuli was not affected suggesting a specific effect for body representation (Blanke et al., 2005). Another experiment showed that, the temporal parietal junction, which is involved in self processing and multisensory integration of body-related information; and the extrastriate body area (EBA), which responds selectively to human bodies and body parts mental imagery is performed with mentally embodied (EBA) or disembodied (TPJ) self location (Arzy et al., 2006). The more intense hallucinations or illusions are usually associated with brain lesions, abnormal brain function such as epilepsy, major psychiatric syndromes, dissociative drugs such as ketamine, or in micro-gravity conditions (Kornilova, 1997).

The study of the lesioned or abnormal brain areas is often used to gain insight into the brain areas involved in normal body representation phenomena. However, there is also anecdotal evidence that these intense hallucinations can occur in non-neurological cases but they have a low occurrence and, apart from micro-gravity illusions, are unpredictable. In the present report, we used functional MRI to examine an otherwise “normal,” healthy individual that reported the ability to, at will, vividly experience her body moving outside her physical body while lying down at rest. The subjective description of the participant led us to use the term extra-corporeal experience (ECE) throughout this manuscript to underline the difference between the phenomenon studied here and the more common definition of out-of-body experiences. We included a number of guided imagery tasks to specify the ECE-related brain activity. One control task was motor imagery for a different movement (jumping jacks). A second control condition was alternating between actual finger movements and motor imagery of the same movement. Finally, we were interested in determining if there was a difference between imagining herself performing the ECE (but not experiencing the ECE) differed from the imagining of another person performing the same ECE movement.

## Materials and Methods

### Participant

The participant was a right-handed woman, age 24, who was a psychology graduate student at the time of testing. She signed an informed consent approved by the University of Ottawa Research Ethics Board. The participant was in an undergraduate class that presented data on body representation hallucinations in patients that report experiences of their body outside their physical body (Blanke and Arzy, 2005). The participant spontaneously reported after class that she could have a similar “out of body” experience. She appeared surprised that not everyone could experience this. The participant described her experience as one she began performing as a child when bored with “sleep time” at preschool. She discovered she could elicit the experience of moving above her body and used this as a distraction during the time kids were asked to nap. She continued to perform this experience as she grew up assuming, as mentioned, that “everyone could do it.” This was often done before sleep onset as an aid to enter sleep. She described the experience as variable depending on her frame of mind. She was able to see herself rotating in the air above her body, lying flat, and rolling along with the horizontal plane. She reported sometimes watching herself move from above but remained aware of her unmoving “real” body. The participant reported no particular emotions linked to the experience. As an adult, the participant only infrequently “practiced” the experience; the experience does not occur spontaneously but is induced wilfully. The participant describes the experience in the following terms: “I feel myself moving, or, more accurately, can make myself feel as if I am moving. I know perfectly well that I am not actually moving. There is no duality of body and mind when this happens, not really. In fact, I am hyper-sensitive to my body at that point, because I am concentrating so hard on the sensation of moving. I am the one moving – me – my body. For example, if I ‘spin’ for long enough, I get dizzy. I do not see myself above my body. Rather, my whole body has moved up. I feel it as being above where I know it actually is. I usually also picture myself as moving up in my mind’s eye, but the mind is not substantive. It does not move unless the body does.”

### Procedure

Four questionnaires were administered. The Pittsburgh Sleep Quality Index (Buysse et al., 1989) was used to detect possible sleep disturbances because sleep onset disturbances have been associated with altered somatosensory or vestibular perceptions (Braithwaite et al., 2011). In order to estimate visual and kinesthetic imagery, the participant was asked to complete the 8-item Movement Imagery Questionnaire-Revised (MIQ-R; Hall and Martin, 1997) and the 20-item Kinesthetic and Visual Imagery Questionnaire (KVIQ; Malouin et al., 2007). Finally, the PAS perceptual aberration scale (Arzy et al., 2007) was administered.

### Data acquisition

The experimenter provided instructions to the participant through MRI earphones. The data was collected in one imaging session during which time both anatomical and functional MR images were obtained. All imaging was performed using a 1.5-T Siemens Magnetom Symphony MRI scanner. The participant lay supine with her head secured in a custom head holder. A conventional T1-weighted spin echo localizer was acquired and used to prescribe a subsequent 3D FLASH (TR/TE 11.2/21 ms, flip angle 60°, field of view (FOV) 26 cm × 26 cm, 256 × 256 matrix, slice thickness 1.5 mm) volume acquisition used for further structural analyses. A T2 FLAIR scan was also performed and inspected by a neuroradiologist following the scanning session to ensure that there was no structural anomaly. Whole brain fMRI was performed using a T2*-weighted echo planar pulse sequence (TR/TE 3000/40 ms, flip angle 90°, FOV 24 cm × 24 cm, 64 × 64 matrix, slice thickness 5 mm, 27 axial slices, bandwidth 62.5 kHz).

Table 1 presents the order and characteristics of each run. The participant was asked after the structural images were acquired if she believed she would be able to “perform” her ECE: she reported being certain she could. Functional imaging runs lasted 59 min in total with an additional 10 min consisting of instructions between runs. Six functional “runs” in the scanner using a block design took place. Runs 1, 4, and 6 involved the participant going in and out of her ECE experience for 5 min at the researcher’s oral command of “start” and “stop.” She induced the ECE to the researcher’s command of “start” and then was stopped after 90 s with the word “stop.” This was repeated four times for Runs 1 and 6 and three times for Run 4. The participant was asked to perform her ECE at the “start” prompt and to tap her finger when she felt herself starting. Prior to imaging she had practiced this tapping at home to ensure it would not interfere with her performance. She was asked to tap her finger again if the ECE stopped before the researcher said “stop.” As this was the case on two trials the blocks were adjusted to maximize the data obtained and the image analysis included scans from the ECE blocks and the rest blocks. If she concluded her ECE prior to the experimenter stopping her she would again tap her finger (in sight of the researchers). In Run 1, the ECE consisted of being above her body and rocking from side-to-side. The participant reported having trouble stopping the rocking movement. The participant also signaled if the movement stopped during the run – the time the movement stopped and re-started was recorded for subsequent analysis. In Run 4, the participant was asked to perform an ECE (above her body and spinning horizontally) and to tap her finger when she felt herself starting. The participant reported difficulty starting the movement (the onset of each sub-run was always delayed contrary to other runs – all timings delays were accounted for in the data analysis). The participant reported that the spinning movement was hard to stop for the rest period. Because the participant in general does not like the spinning movement (she gets dizzy), she switched to a “bobbing on the ocean” movement during Run 4 and informed the experimenter after the end of that run. In Run 6, the ECE was the bobbing movement: the participant reported the sub runs as being less “sharp.”

**Table 1:** **Characteristics of each run during the scanning session**.

Run number	Blocks	Number of scans (TR = 3 s)	Block durations
1	Extra-corporeal experience 1 vs. rest	200	ECE 90, 87, 96, 108 s
			Alternating rests of 60 s between each ECE block
2	Visualizing ECE in someone else vs. self	103	30 s Alternating blocks
3	Jumping jacks vs. rest: kinesthetic imaging	103	30 s Alternating blocks
4	Extra-corporeal experience 2 vs. rest	200	ECE 90, 87, 96, 108 s
			Alternating rests of 60 s between each ECE block
5	Actual finger movement vs. visualization	103	30 s Alternating blocks
6	Extra-corporeal experience 3 vs. rest	200	ECE 66, 60, 87 s
			Alternating rests of 60 s between each ECE block

The second, third, and fifth runs were guided motor imagery. Run 2 included an experimenter instructing with one word (either “someone” or “you”) every 30 s, alternating while she *visualized* (but not *experienced*) herself actually moving as she did in the ECE or while she *visualized* someone else doing the same movement. This was a 5-min task. The informal comment from the participant was that she did not “feel herself moving” when “visualizing” her experience during run 2. We were interested in determining if there was a difference between imagining herself performing the ECE (but not experiencing the ECE) differed from the imaging of another person performing the same ECE movement. Run 3 included the same alternating block design whereby the participant imagined herself *performing* jumping jacks or *resting*: this was a control task to determine which structures were involved in non-ECE motor imagery. The participant practiced the instructions for Run 3 prior to starting the run to ensure that she was able to visualize herself. From the participant’s comments, it was inferred that visualizing herself doing jumping jacks did not involve the movement sensations associated with her extra corporeal experience. Run 5 involved the participant *moving* her right hand fingers (one at a time) to her thumb at a frequency of 2 Hz and then *visualizing* herself perform the same movement. Again, the participant did not report a sensation of movement. This control task was added to determine the brain areas involved in a simple motor action and its imagined version. Again, each block was 30 s and the Run was 5 min. Our conversations with the participant suggested that her extra corporeal experience involved the sensation of movement while other imagery tasks she performed did not involve this sensation.

### Image post-processing

The functional images were reconstructed and whole brain images were realigned to correct for motion by employing the procedure of Friston et al. (1995), using Statistical Parametric Mapping (SPM8) software. The motion correction did not exceed 1 mm. Images were spatially normalized to match the echo planar imaging (EPI) template provided in SPM8 with 2 mm × 2 mm × 2 mm voxel sizes. Images were then smoothed with a 10 mm full-width at half-maximum Gaussian filter.

### Statistical analyses

A fixed effects analysis was performed with data from each Run separately. The blocks of ECE were compared with the rest blocks from the same Run. The Runs with motor imagery and/or visualizations were analyzed by contrasting the two types of blocks, for example in Run 3 scans from the rest blocks were subtracted from the visualization of jumping jack blocks (Jumping Jacks minus Rest).

## Results

### Questionnaires

The MIQ-R results indicated that the participant had kinesthetic imagery comparable to that observed in competitive sport athletes (*M* = 5.5) but higher visual imagery (*M* = 7) (Roberts et al., 2008). In the KVIQ, the participant scored an average of 4.1 on the visual imagery scale (comparable to healthy but older controls) and 4.3 on the kinesthetic imagery scale, which is higher than the same controls. The Pittsburgh Sleep Quality Index (PSQI = 5) was slightly higher than would be expected in healthy participants (PSQI = 2.67): this was essentially due to longer sleep latency (90 min). In the PAS perceptual aberration scale, the participant responded “false” to most statements except for the following items (her answers in italics): (T.12) Now and then, when I look in the mirror, my face seems quite different than usual. (*Only when contemplating my own mortality*); (T.15) Sometimes when I look at things like tables and chairs, they seem strange. (*Occasionally but voluntary*. *Sometimes late at night, I can play with perspective i.e., make things appear closer/farther away. Also, sometimes, ordinary objects seem bizarre in the sense that all existence is bizarre*); (T.23) It has seemed at times as if my body was melting into my surroundings. (*Always voluntary*. *I can make it feel like my body is going down into my bed*); (T.31) Sometimes I feel like everything around me is tilting. (*Almost always this is voluntary* … *usually when I am bored in class*).

### ECE results

The participant reported being successful at beginning and ending her ECE on demand of the experimenter. The experience for Run 1 began immediately and she began to see herself above her body rocking with her feet moving down and up as her head moved up and down as in bobbing in ocean waves. The second ECE Run was the most intense and involved the participant watching herself above her own body, spinning along the horizontal axis. The final ECE involved the participant spinning as in the second ECE.

**Figure 1:**
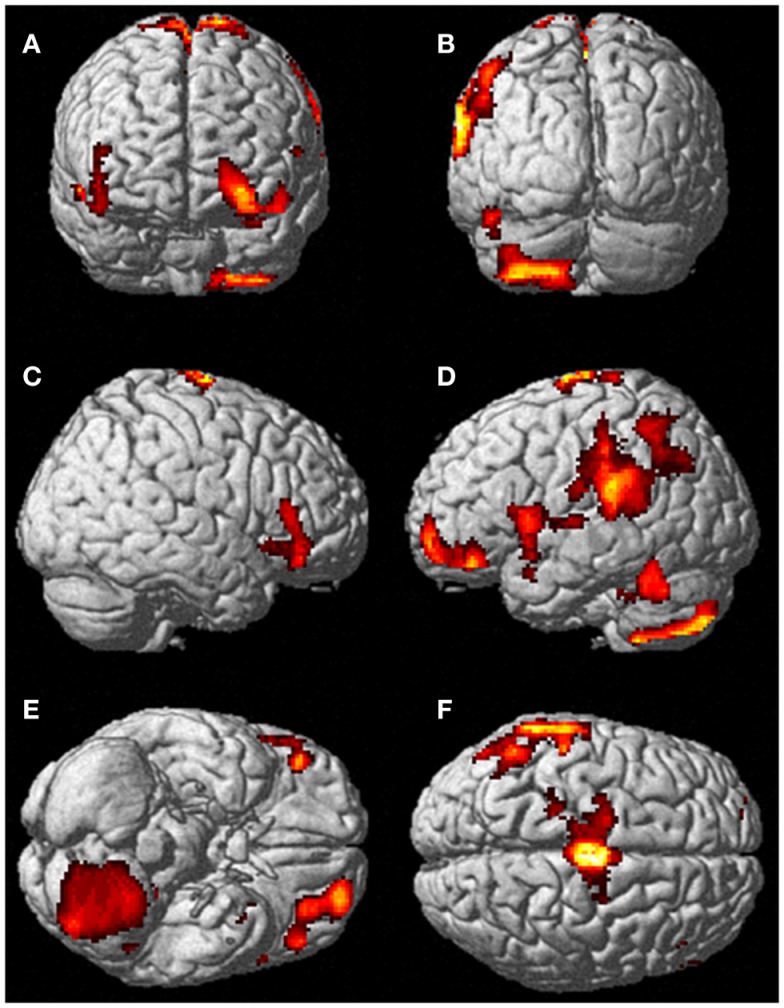
**Rendered image of significantly activated regions of the brain while the participant was having extra-corporeal experiences**. Most significantly activated regions are lateralized to the left side and include the supplementary motor area **(F)**, the cerebellum **(B,D,E)**, the supramarginal gyrus **(D,F)**, the inferior temporal gyrus **(B,D,F)**, the middle and superior orbitofrontal gyri **(A,C,D,E)**. The *p*-value was set at 0.001 uncorrected for this image with the cluster threshold at 200 significant voxels.

**Figure 2:**
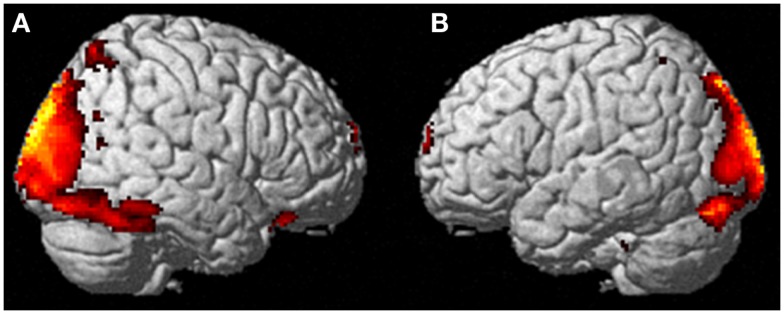
**Areas of reduced activity during the ECEs compared to rest**. The visual cortex is particularly impacted. **(A)** Representation of the right side; **(B)** activity on the left. The *p*-value for this image was set at 0.05 FWE corrected.

Neural activation patterns for each of these ECE Runs were analyzed separately with rest subtracted from the experience. Given the lack of significant difference between the results of each of the three Runs, all ECE Runs were combined into one analysis to increase power and observe brain regions that were concomitantly activated for each Run. Results are reported with a family wise error (FWE) very stringent correction for multiple comparisons at 0.001. Results are presented in Figure 1. The most significantly and consistently activated areas during the ECE compared to the non-ECE blocks were left lateralized in the supplementary motor area (SMA) (*x*, *y*, *z* = −2, −18, 62, cluster 247, *T* = 6.66, *p* = 0.001), supramarginal gyrus/posterior superior temporal gyrus (*x*, *y*, *z* = −64, −46, 24, cluster 60, *T* = 6.04, *p* = 0.001), inferior temporal gyrus (*x*, *y*, *z* = −48, −54, −20, cluster 72, *T* = 5.89, *p* = 0.001), middle and superior orbital frontal gyri (*x*, *y*, *z*, = −26, 56, −10, *T* = 5.05, *p* = 0.001), and the cerebellum (*x*, *y*, *z* = −50, −48, −30, *T* = 5.76, *p* = 0.001). The parietal and superior temporal activation taken together correspond to the temporal parietal junction. There was significantly less activation during the ECE blocks compared to non-ECE blocks (Figure 2) in bilateral posterior visual regions: the lingual gyrus (*x*, *y*, *z* = 14, −64, 4, cluster 19205, *T* = 13.23, *p* = 0.001) and the cuneus (*x*, *y*, *z* = 0, −92, 18, cluster 19205, *T* = 12.71, *p* = 0.001).

### Visualization results

**Figure 3:**
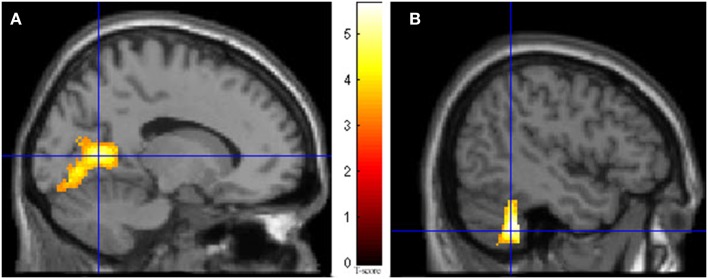
**Results from visualizing herself doing the same action she performed in the first ECE vs. visualizing another person performing the same movement**. **(A)** Bilateral lingual gyrus differences in activity and **(B)** the left cerebellar differences. The *p*-value for this image was set at 0.001 uncorrected.

During imagining herself moving as she did in the first ECE (Run 1), without inducing an ECE, controlling for multiple comparisons at a *p* < 0.001, the participant activated more left cerebellum (*x*, *y*, *z* = −46, −48, −44, cluster 406, *T* = 5.66, *p* = 0.001) and bilateral lingual gyrus (*x*, *y*, *z* = −14, −62, 6, cluster 980, *T* = 5.00, *p* = 0.001; *x*, *y*, *z* = 6, −58, 8, cluster 790, *T* = 4.82, *p* = 0.001) than when imagining someone else moving in the same way (Figure 3). Similarly, she showed significantly less activity during self-imagining than imagining someone else in the bilateral superior orbital frontal gyrus (*x*, *y*, *z* = −18, 66, −2, cluster 148, *T* = 4.40, *p* = 0.025; *x*, *y*, *z* = 14, 68, −2, cluster 146, *T* = 4.38, *p* = 0.026).

**Figure 4:**
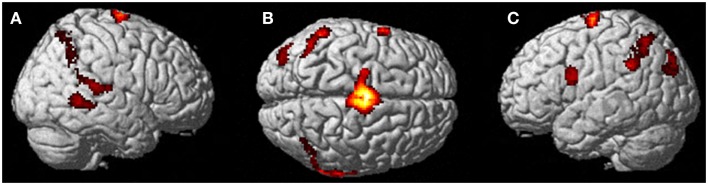
**Results from visualizing herself performing jumping jacks compared to rest**. **(A)** Right hemisphere; **(B)** dorsal view of the SMA activity; and **(C)** left hemisphere activation. The *p*-value for this image was set to 0.001 uncorrected with the cluster threshold at 100 significant voxels.

The second control task involved the participant imagining herself performing jumping jacks and then not imagining anything and just keeping her eyes closed waiting for the next start cue for the jumping jacks. Results are presented in Figure 4. The imagining of herself performing the jumping jacks, controlling for multiple comparisons at *p* < 0.001, revealed significantly more activity in the posterior SMA (*x*, *y*, *z* = −2, −10, 60, cluster 1424, *T* = 7.95, *p* = 0.001), paracentral lobule (*x*, *y*, *z* = 0, −12, 68, cluster 1424, *T* = 6.72, *p* = 0.001), middle temporal gyrus (BA22) (*x*, *y*, *z* = 68, −48, 8, cluster 132, *T* = 5.72, *p* = 0.04), precentral gyrus (BA44) (*x*, *y*, *z* = −60, 6, 22, cluster 136, *T* = 5.11, *p* = 0.035), inferior parietal lobule (*x*, *y*, *z* = −40, −64, 58, cluster 265, *T* = 4.64, *p* = 0.001), and superior temporal gyrus (BA22) (*x*, *y*, *z* = 68, −34, 12, cluster 156, *T* = 4.78, *p* = 0.019). The TPJ activity was more bilateral than during the ECE runs (Figure 4). There was also less activity in bilateral cuneus (*x*, *y*, *z* = 6, −76, 4, cluster 22067, *T* = 10.16, *p* = 0.001) and bilateral superior orbital frontal gyrus (*x*, *y*, *z* = −28, 26, −28, cluster 617, *T* = 6.50, *p* = 0.001; *x*, *y*, *z* = 4, 48, −28, cluster 455, *T* = 5.69, *p* = 0.001) during the jumping jack imagery compared to rest.

**Figure 5:**
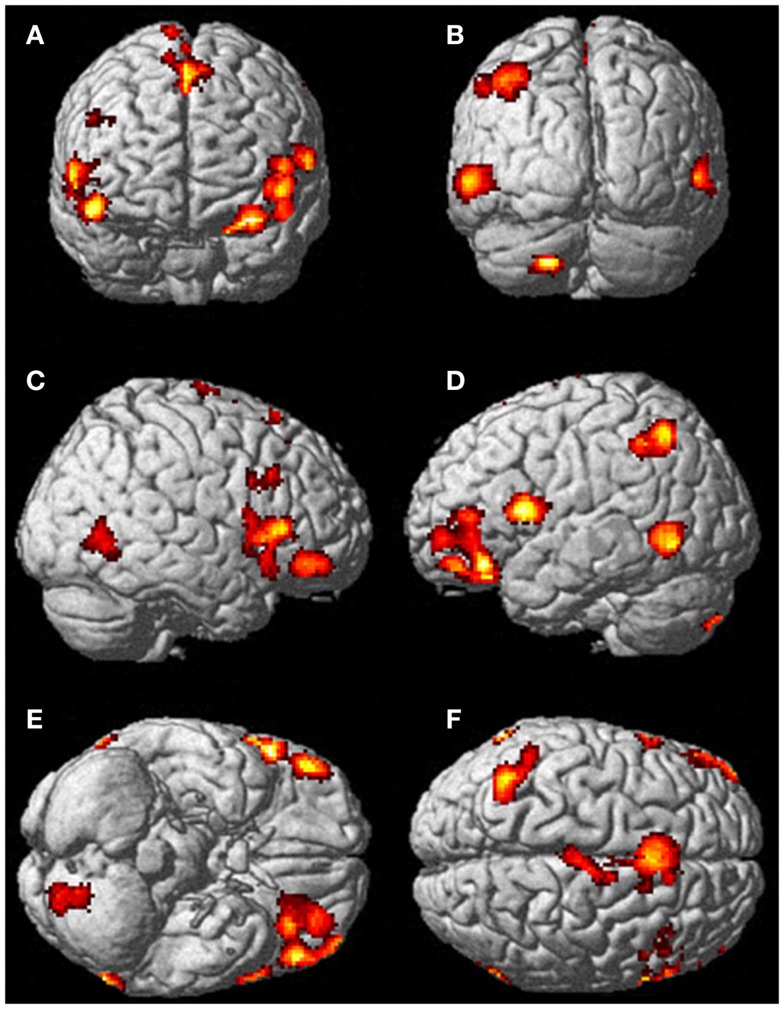
**There was significantly more activation during the visualization of finger movement compared to the actual movement**. Each letter represents a different view of the brain **(A)** anterior view, **(B)** posterior view, **(C)** right lateral view, **(D)** left lateral view, **(E)** ventral view, and **(F)** dorsal view. The *p*-value for this image was set to 0.001 uncorrected with the cluster threshold at 100 significant voxels.

**Figure 6:**
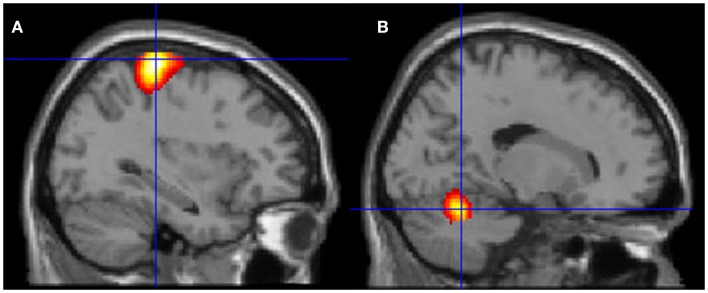
**Motor areas significantly activated more during movement of her fingers to thumb compared with visualizing the same movement**. **(A)** Representation of the left primary motor cortex; **(B)** representation of the right cerebellum. The *p*-value for this image was set to 0.001 uncorrected with the cluster threshold at 100 significant voxels.

Another contrast of interest was the actual movement of the fingers to the thumb compared with imagining the same movement (Figure 5). There was significantly more activation during the imagining vs. the actual movement in several areas that were similarly (but not identically) activated during the ECE. These included the bilateral inferior frontal triangularis (*x*, *y*, *z* = 50, 40, −14, cluster 326, *T* = 5.27, *p* = 0.001; *x*, *y*, *z* = −42, 58, 0, cluster 1132, *T* = 5.18, *p* = 0.001), left middle temporal gyrus (*x*, *y*, *z* = −62, −58, −2, cluster 371, *T* = 6.31, *p* = 0.001), left cerebellum (*x*, *y*, *z* = −22, −88, −46, cluster 270, *T* = 5.97, *p* = 0.002), left superior parietal lobule (*x*, *y*, *z* = −36, −60, 50, cluster 581, *T* = 5.56, *p* = 0.001), and a more anterior part of the SMA (bilateral) (*x*, *y*, *z* = 0, 14, 58, cluster 711, *T* = 5.56, *p* = 0.001). Finally, there was significantly less activity during imagining than movement (Figure 6) in the left postcentral and precentral gyri (*x*, *y*, *z* = −32, −30, 70, cluster 1756, *T* = 12.85, *p* = 0.001; *x*, *y*, *z* = −36, −30, 62, cluster 1756, *T* = 12.05, *p* = 0.001, respectively), and right cerebellum (*x*, *y*, *z* = 10, −56, −22, cluster 997, *T* = 9.95, *p* = 0.001), areas similar to those activated during the jumping jack condition.

## Discussion

The present experiment examined functional brain imaging patterns in a participant that reported being able, at will, to produce somatosensory sensations that are experienced as her body moving outside the boundaries of her physical body while remaining aware of her unmoving physical body. It is interesting that the development of the participant’s ability was associated with delayed sleep onset in childhood (which persisted in adulthood) because the occurrence of out-of-body experiences has been frequently associated with hypnagogic phenomena (Cheyne et al., 1999; Terhune, 2009). The reported experience is similar to what is defined by Brugger as an out-of-body experience but without the feeling of being only outside of her body and without any of the emotional content typically reported in out-of-body experiences (Brugger and Regard, 1997). The subjective description of the participant led us to use the term ECE throughout this manuscript to underline the difference between the phenomenon studied here and the more common definition of out-of-body experiences. Also, because the ECE was private to the participant, we have to rely on the participant’s descriptions to interpret the results. With these caveats in mind, we find that the brain functional changes associated with the reported ECE were different than those observed in motor imagery. The results suggest that the ECE reported here represents an unusual type of kinesthetic imagery that shares some features of previously described out-of-body experiences and some features of more typical motor imagery.

The ECE was reported as a mixture of visual imagery and kinesthetic imagery but the kinesthetic component was prominent as evidenced by the report of feeling dizzy when performing a rotational movement. The prominence of kinesthetic experience over the visual experience is consistent with a strong bilateral deactivation of the lingual gyrus and cuneus encompassing the primary visual cortex. Activations are mainly left-sided and involve the left SMA, supramarginal and posterior superior temporal gyri (the last two overlap with the temporal parietal junction, which has been associated with out-of-body experiences). The cerebellum also shows strong activation that is consistent with the participant’s report of the impression of movement during the ECE. There are also left middle and superior orbital frontal gyri activations, structures often associated with action monitoring.

The TPJ activation that was observed during the ECE is consistent with patient cases that report autoscopy and out-of-body experiences when the functional integrity of that area is altered (Blanke et al., 2004; Blanke and Mohr, 2005; Blanke, 2012). Studies of experimentally induced altered body imagery have demonstrated that transcranial magnetic stimulation of the TPJ area can interfere with the ability of healthy individuals to imagine themselves in body orientations similar to out-of-body experiences (Blanke et al., 2005). Electrical stimulation of the TPJ in epileptic patients also produces various sensations associated with out-of-body experience (Blanke et al., 2002). Interestingly, several of the active clusters found in the present experiment during the ECE (left supramarginal gyrus, left inferior temporal gyrus, left cerebellum) correspond closely to clusters with mirror properties associated with action observation and execution that were identified by a recent meta-analysis (Molenberghs et al., 2012).

The middle orbital frontal gyrus is a highly multimodal area that has been associated with performance monitoring and provides flexibility in response to selection based on ongoing feedback (Elliott et al., 2000). The cluster that we observed in the left orbital frontal gyrus corresponds to cluster 6 of the K-6 solution described by (Kahnt et al., 2012) in their parcelation of the orbitofrontal cortex (Kahnt et al., 2012). They reported functional connectivity with adjacent regions in the lateral prefrontal cortex as well as regions in the inferior parietal cortex and the lateral inferior temporal cortex; the latter two structures correspond to activations we observed during the ECE.

We also instructed the participant to alternate between visualizing herself performing her ECE and visualizing someone else performing the same movement with the specific instruction that she should not experience the ECE but only “see” it. The goal was to guide the participant toward taking a first-person perspective of her own experience and transposing it to a third-person perspective. The first-person perspective was associated with a bilateral increase in the lingual gyrus and another one in the left cerebellum: this may indicate that imagining herself included both a visual component and possibly a kinesthetic component (even following a specific instruction to avoid this) that was absent when visualizing using the third-person view. The self-visualization was accompanied by a reduction in orbitofrontal activation that may indicate that visualizing herself was easier than taking the third-person view and required less monitoring of activity. Jackson et al. (2006) studied activations in participants observing hand or foot movements seen either from a first-person perspective or a third-person perspective. They found significantly more activity in the left sensory-motor cortex for first-person, during observation alone, and in the lingual gyrus for third-person perspective suggesting that perspective taking is associated with a different pattern of activation (Jackson et al., 2006). It is difficult to reconcile the higher lingual cortex activity observed with our participant taking the first-person view and the higher activity with the third-person perspective in Jackson et al. (2006). However, in that study, participants were only shown pictures corresponding to first- or third-person view of static limbs whereas our participant was instructed to visualize a whole body movement. A similar procedure contrasting first and third-person view was used in a study in which participants viewed hand movements from the two perspectives (Lorey et al., 2009) and in a study where participants were instructed to imagine using a tool presented to them on a picture or imagine someone else using the same tool (Ruby and Decety, 2001). Both these studies reported activation differences when contrasting first- and third-person views. Our results obtained comparing first- and third-person perspective for the ECE experience is similar in that activation differences were observed between the two conditions when the participant “only imagined” the ECE. The pattern of differences that we observed was unsurprisingly quite different than in previous studies likely owing to the task differences and the number of participants (Ruby and Decety, 2001; Lorey et al., 2009).

In the third condition, we examined the brain areas involved in a whole body motor imagery to examine if the ECE was similar to motor imagery in this participant. The first general observation is that in this condition, activations tended to be bilateral as opposed to mainly left-sided activations observed in the ECE. The second observation is that the activations when the participant was told to imagine doing jumping jacks were less extensive than for the ECE. They included bilateral SMA extending into the paracentral lobule, bilateral inferior parietal lobule, right middle and superior temporal gyri, and left precentral gyrus. There was reduced activity in the cuneus bilaterally and in the superior orbital frontal gyrus also bilaterally. Activations of the SMA, inferior parietal lobule, and precentral gyrus have been reported in two previous studies of kinesthetic imagery using hand movements (Guillot et al., 2009; Szameitat et al., 2012b). ECE and whole body motor imagery were both associated with a reduction in cuneus activation (but less so for motor imagery) suggesting that visual imagery was inhibited during both conditions. During motor imagery, there was less activity in the superior orbital frontal cortex whereas there was more activity in the middle and superior orbital frontal cortex during ECE. This is suggestive of more motor monitoring during ECE than motor imagery.

The last condition was an attempt to compare the activations associated with actual hand movements to imagining the same movement in this participant (Guillot et al., 2009; Szameitat et al., 2012b). In one of these studies, there were 13 participants selected on the basis of excellent motor imagery (Guillot et al., 2009) whereas the other included 21 unselected participants (Szameitat et al., 2012b). The number of participants in both these studies achieved a greater statistical power and reported many more activations than in the present single-case study. The finger movements used in the Guillot et al. study was a learned and practiced sequence, more complex than the one we used, which could be considered more of an automatic nature. The movement used in the Szameitat et al. study consisted of a simple wrist movement timed with a tone. Although it is not clear how comparable these studies are with the present observations, there are a number of concordant findings. First, real and imagined movements produce activations in the SMA. The activations reported by Szameitat et al. (2012a,b) in the contrast imagery-rest include premotor areas in the precentral gyrus, superior frontal gyrus, and bilateral inferior frontal gyri that were also observed in the “jumping jacks” condition of our participant.

It has been shown that visual imagery is reliant on the occipital lobe and the superior parietal lobule, as well as lateral premotor cortex, while kinesthetic imagery is more associated with motor areas and inferior parietal activity (Guillot et al., 2009, p. 698). The ECE in the present study activated the left side of several areas associated with kinesthetic imagery and was associated with a strong deactivation of the visual cortex. This suggests that her experience really was a novel one, with a strong kinesthetic component. This was a healthy young woman with no brain abnormalities, thus providing a window into the brain during non-pathological, self-elicited ECE.

There are a number of limitations to the present study. The first obvious one is that we relied on the participant’s report of her experience. Given that the participant spontaneously reported her experience assuming that it was a common occurrence and the detailed (and unusual) description of how she developed this ability, we are inclined to take her report at face value. The private nature of imagery is common to most research in imagery (including other imagery conditions in the present report) although a number of control measures have been devised but they were not used here. One example of such measures is the increase in heart rate and pulmonary ventilation during imagined actions (Decety et al., 1993; Wuyam et al., 1995). The description of the imagery tasks could have been more clearly specified including the “jumping jacks” condition and the third-person ECE task (Moran et al., 2012). Statistical power was obviously limited in this single-case study, which means that potentially several activations escaped detection. Limited statistical power could also have prevented us from finding activation differences when the participant performed “variations” of her ECE experience (spinning vs. “bobbing on the ocean”).

This is the first study with a non-pathological participant who is able to elicit an ECE upon demand. Clearly, replication is required to ascertain if this pattern of activation is similar in other people who can have self-initiated ECE. The existence of such a case and its presentation raises the possibility that this phenomenon may have a significant incidence but unreported because people do not think this is exceptional. Alternatively, the ability might be present in infancy but is lost without regular practice. This would be reminiscent of the discovery and eventual study of synesthesia that some researchers now hypothesized is more prevalent in young people or can be developed (Deroy and Spence, 2013; Simner, 2013).

## Author Contributions

Claude Messier and Andra M. Smith designed the experiment, collected the data, and wrote the manuscript. Andra M. Smith analyzed the MRI data and prepared the figures.

## Conflict of Interest Statement

The authors declare that the research was conducted in the absence of any commercial or financial relationships that could be construed as a potential conflict of interest.
